# Brain fag syndrome: a culture-bound syndrome that may be approaching extinction

**DOI:** 10.1192/pb.bp.114.049049

**Published:** 2015-08

**Authors:** Oyedeji A. Ayonrinde, Chiedu Obuaya, Solomon Olusola Adeyemi

**Affiliations:** 1 South London and Maudsley NHS Foundation Trust, London, UK; 2 Camden and Islington NHS Foundation Trust, London, UK; 3 Federal Neuro-Psychiatric Hospital, Kaduna, Nigeria

## Abstract

**Aims and method** To explore the current salience of ‘brain fag’ as a nosological, diagnostic and clinical construct in modern West African psychiatry. A semi-structured questionnaire and vignette based on classical symptoms of brain fag syndrome were used to explore current knowledge, explanatory models and practice among Nigerian psychiatrists.

**Results** Of 102 psychiatrists who responded, 98% recognised the term ‘brain fag syndrome’ and most recognised the scenario presented. However, only 22% made a diagnosis of brain fag syndrome in their practice preferring diagnoses of anxiety, affective and somatic disorders.

**Clinical implications** A decreasing number of Nigerian psychiatrists are making a diagnosis of ‘brain fag syndrome’. We found strong evidence of nosological and diagnostic decline in the syndrome in its place of birth. This may signal the early extinction of this disorder or nosological metamorphosis from a ‘culture-bound’ syndrome in West African psychiatric practice.

In 1960 Raymond Prince,^1^ a founding figure in the field of transcultural psychiatry, published a seminal paper describing unique cultural observations in Nigeria, a newly independent country in British West Africa. At the time, Prince (a Canadian psychiatrist) had been working in Yoruba-speaking south-western Nigeria. In the paper he described a cluster of symptoms he observed in Nigerian students, which were associated with study and manifesting a range of emotional and somatic complaints. Given the recurrent reference to mental exhaustion (‘brain fag’) Prince named this symptom cluster ‘brain fag syndrome’.

Distinctive symptoms of the syndrome were:

- intellectual impairment
- sensory impairment (chiefly visual)
- somatic complaints, most commonly of pain or burning in the head and neck
- other complaints affecting the student's ability to study
- an unhappy, tense facial expression
- a characteristic gesture of passing the hand over the surface of the scalp or rubbing the vertex of the skull.

Citing Prince:

‘Most often the symptoms commenced during periods of intensive reading and study prior to examinations or sometimes just following periods of intensive study … The patients generally attribute their illness to fatigue of the brain due to excessive “brain work”.’ (p. 561)^1^

As Price elaborated, it is for this reason that the term brain fag syndrome has been used. The expression seems to be used by Nigerians generally to describe any psychiatric disturbance occurring in ‘brain workers’. Exploring the unfamiliar nature of the presentation, brain fag was at the time ascribed to ‘a clash between Western and African cultural values’.^1^

This unfamiliar clinical presentation and manifestation of distress was initially felt to be a culture-bound syndrome localised to southern Nigeria. Subsequently, further reports were published from other African regions.^2,3^ These case reports and student population surveys highlighted psychological distress associated with study in some African students.^2,3^ Over the decades, there has been some debate by predominantly African psychiatrists as to whether this actually constitutes a unique nosological entity, cultural manifestation or unique amalgamation of more established mental disorders.^4-6^ An interesting review of brain fag syndrome literature by Ola *et al*^5^ details the different arguments.

While reference is made to brain fag syndrome in both the tenth edition of ICD-10^7^ and the ‘Glossary of Culture Bound Syndromes’ in the fourth edition (revised) of DSM-IV-TR,^8^ the current relevance to clinical practice is uncertain. The DSM IV-TR described brain fag syndrome as a culture-bound syndrome attributed to overwork and affecting West African students. Characterised by a loss of ability to concentrate, learn, remember or think and usually accompanied by sensations of pain, pressure or tightness around the head or neck and blurred vision, a condition which ‘can resemble certain Anxiety, Depressive and Somatoform Disorders’.^8^ The newer DSM-5, however, is less detailed regarding brain fag and cites it as an example of a cultural anxiety syndrome.^9^

Nigerian psychiatric services, although based on Western models, are also familiar with the assessment and treatment of West African cultural presentations of affective, psychotic and anxiety disorders. Competition with traditional and faith healers abounds as do belief systems about emotional distress. The ICD-10 is primarily used for diagnostic classification, although DSM classification is also recognised. Medical instruction and scholarship is in English and the majority of practitioners are at least bilingual and speak one or more indigenous languages.

The limited diagnostic clarity about the validity of this culture-bound syndrome^5^ justifies an exploration of the existence of brain fag in modern West African psychiatry. A factor analysis and differentiation of somatic symptoms in Africans with depression^10^ and the validation of burning and crawling sensations in the Brain Fag Syndrome Scale are important empirical steps towards this.^11^ If a prevalent disorder, the exponential growth in secondary and tertiary educational institutions (online Fig. DS1) would potentially have an impact on the mental well-being of millions of adolescents and young adults with health and resource implications. Conversely, if the diagnostic status is unclear, the pathologisation of young people may lead to inappropriate help-seeking or impair education.

When brain fag syndrome was originally described in Nigeria in 1960, Western models of psychiatric care were in their infancy, with only four indigenous, linguistically and culturally embedded psychiatrists in the country. International psychiatrists from the UK, the USA, Canada (including Raymond Prince) and Germany made significant contributions to service development and the training of mental health professionals in the country. While the number of mental health professionals has shown sustained growth over the years, the number of psychiatrists remains grossly inadequate, with an estimated 0.15 psychiatrists per 100 000 Nigerians.^10^ Unfortunately, 25% migrate within 5 years of completing training.^12^ Such specialist workforce changes may influence skill stability.

There have been no known surveys to date of West African mental health professionals on their opinions and practice relating to brain fag syndrome.

## Aim

This study aimed to explore awareness of brain fag, its aetiology, diagnosis, explanatory models and management by psychiatrists in Nigeria, where this disorder was originally described.

We sought to explore the nosological salience of brain fag syndrome among contemporary West African psychiatrists in Nigeria and to ascertain whether it is still regarded as a distinct disorder or an idiomatic expression. We were also interested in exploring the historical course of the syndrome over time. Opportunities to explore the modern and cultural history of disorders do not arise often in anthropological medicine.

## Method

A semi-structured, self-administered questionnaire that incorporated both coded and written text responses was designed as a survey of ‘psychological distress’ among students. It was mailed to psychiatrists in tertiary, secondary and private psychiatric facilities across Nigeria. Electronic copies were also disseminated. Further uptake was facilitated at the conference of the Association of Psychiatrists in Nigeria, the key professional body for psychiatrists. Questions were clustered into the following categories:

1. clinical experience in psychiatry (number of years of practice, seniority)
2. setting of clinical practice (e.g. teaching hospital, specialist psychiatric hospital, general hospital and private practice)
3. geographical region of country
4. clinical case-load and number of patients seen per month.

Respondents were presented with a vignette (Box 1) describing classic brain fag features in a student. Though based on core diagnostic features of brain fag syndrome, the term ‘brain fag’ was withheld from respondents until the end of the questionnaire. Respondents were asked a few short questions (e.g. ‘Are you familiar with this type of presentation in your practice?’), the frequency and approximate number of patients with this unnamed symptom cluster they had seen in the past 12 months. It is worth stressing that these questions referred to symptoms in the vignette and not a specific diagnosis of brain fag syndrome.

**Box 1** Vignette presented to psychiatrists in the study

A 20-year-old student presents complaining of ‘burning heat or pain in the head and neck’.The student also experiences difficulty concentrating, assimilating and recalling things studied, as if ‘the brain … is dead or not working’.Vision is sometimes blurred. Sleep has also been poor.The student wishes to pursue further studies and is concerned about the implications of this experience.

From the vignette, the psychiatrists were then asked to consider a diagnosis and differential diagnosis as they would in routine clinical practice such as anxiety disorder, depressive disorder, somatisation disorder, psychotic disorder, or another category. In addition, they were asked to proffer aetiological explanations for the presentation as well as the therapeutic options that they would offer the individual in the scenario. This was a free-text section so as to allow folk, social and other non-medical explanatory models. A content analysis of these responses was carried out to identify themes.

The concept of brain fag syndrome was introduced in the final part of the questionnaire to minimise contamination or bias to the earlier responses. This section explored whether the respondent had ever heard of brain fag syndrome and whether they made this diagnosis in routine clinical practice. The psychiatrists were encouraged to provide additional comments or alternative explanations, views and opinions on any aspect of the study.

Questionnaires were returned by prepaid envelope, hand delivery and electronically.

In addition, five decades of historical context into psychiatric services in Africa and the genesis of brain fag syndrome were obtained through personal communication with current and retired psychiatrists, as well as an anthropological field worker and interpreter used in the original work. Full qualitative details are outside the scope of this paper, but are being prepared for publication in another paper.

## Results

Overall, 102 responses were received from all regions of the country. The national response rate of 36%, while relatively low, overcame logistical challenges and showed a good geographical, ethnic and clinical spread across Nigerian mental health services. Interestingly, this rate mirrored a mail survey of psychiatrists by the World Health Organization and the World Psychiatric Association (Nigeria 36%, USA 21%, UK 22%, South Africa 24%) across 44 countries^13^ and was also similar to other non-incentivised physician surveys.^14^

So as not to restrict opinions, more than one diagnostic response was permitted to some questions, therefore some totals were over 100%.

### Psychiatrist characteristics

**Fig. 1:**
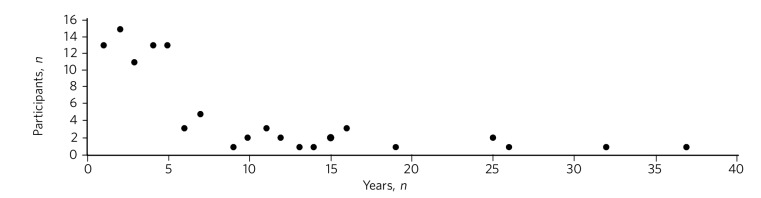
Distribution plot of the respondents' number of years' experience in psychiatry

Forty-four percent of respondents were registrars, 33% senior registrars, 21% consultants and 2% psychiatric medical officers. The mean number of years of experience in psychiatry was 6.3 with a median of 4 years and a range of 1-37 years (Fig. 1). The doctors saw an average of 152 patients per month.

### Location of practice

Sixty-nine (68%) of the psychiatrists were from the south of Nigeria and 33 (32%) were from the northern regions. This distribution closely reflects the regional density of psychiatric services in the country.

### Awareness of symptoms in the brain fag questionnaire vignette

**Fig. 2:**
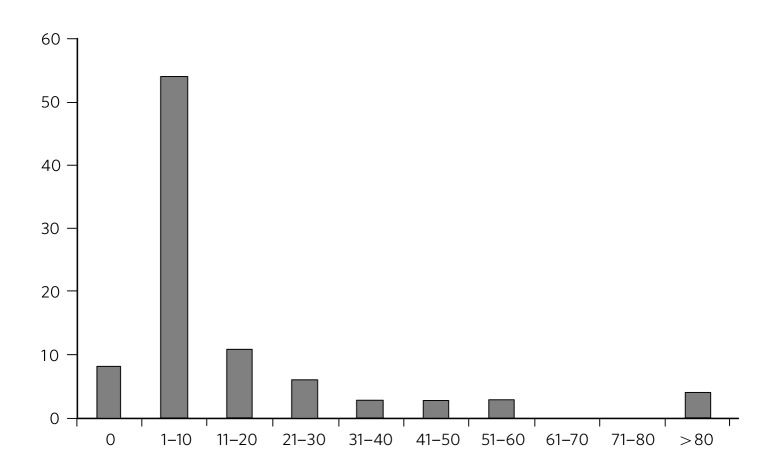
Number of patients with brain fag symptoms seen in past year

The majority of psychiatrists (95%) were familiar with the presentation in the vignette in their routine practice and 84.3% (*n* = 86) had seen patients with similar symptoms within the past year (Fig. 2). On average, each psychiatrist saw 14.2 patients presenting with vignette symptoms annually, approximately 0.78% of their annual clinical case-load or 1:128 patients.

### Nosology of symptoms and signs

The vignette symptoms were classified as an anxiety disorder by 49% of psychiatrists, while 37% considered brain fag syndrome; 36% felt the presentation was consistent with a depressive disorder and 30% associated it with somatisation disorder.

### Aetiological explanations and attributions

Nigerian psychiatrists suggested a number of possible aetiological factors for the brain fag syndrome core criteria vignette symptoms (Table 1). Content analysis observed explanatory themes attributed to the following causes: mental disorders (9.23%), psychogenic (24.10%), educational difficulties (32.31%), sociocultural (9.23%), interpersonal (3.59%), biological (9.74%), substance misuse (4.61%) and demographic factors (1.02%) while 6.15% of the psychiatrists gave no aetiological explanation.

**Table 1:** Causes of vignette symptoms suggested by respondents (*n* = 195)

Category	Subcategory	*n*
Mental disorder (*n* = 18, 9.23%)	Somatisation	14
	Anxiety	2
	Depression	1
	Psychiatric history	1

Psychogenic (*n* = 47, 24.10%)	Stress	29
	Psychological	12
	Poor coping mechanisms	3
	Behavioural	2
	Loss	1

Study and education (*n* = 63, 32.31%)	Educational concerns	31
	Influence of foreign language	10
	Intensive study	9
	Desire to succeed	7
	Fear of failure	2
	Goal failure	1
	Motivational factors	1
	Low productivity	1
	Modernisation through study	1

Sociocultural (*n* = 18, 9.23%)	Socioeconomic	11
	Cultural	3
	Environmental	3
	Witchcraft	1

Substance misuse (*n* = 9, 4.61%)	Psychostimulants	

Interpersonal issues (*n* = 7, 3.59%)	Personality	4
	Strained relationships	1
	Family disharmony	2

Organic/biological (*n* = 19, 9.74%)	Genetic	13
	Biological	2
	Sympathetic activity	2
	Trauma	1
	Malaria	1

Demographic (*n* = 2, 1.02%)	First born	1
	Male gender	1

Unknown (*n* = 12, 6.15%)

### Management

**Fig. 3:**
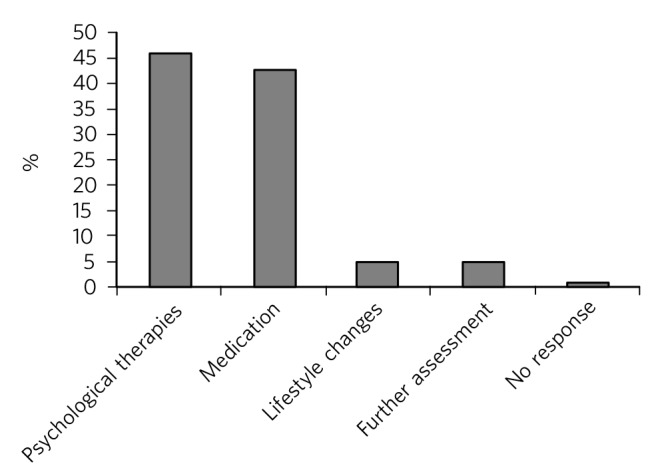
Management of brain fag symptoms

Nearly half of the psychiatrists (46.47%) advocated the use of psychological therapies such as psychotherapy, counselling, cognitive-behavioural therapy and family therapy in the management of the symptoms. Psychotropic medications (e.g. antidepressant, anxiolytics) were suggested by 42.75%. Lifestyle changes such as changing study methods and sleep hygiene were recommended by 4.83% of the respondents (Fig. 3).

### Recognition of brain fag syndrome

Ninety-eight percent of the psychiatrists surveyed had heard of brain fag syndrome. However, only 22% of them actually made this diagnosis in their daily clinical practice.

There was a significant association between the duration of practice in mental health services and the diagnosis of brain fag syndrome (*P* = 0.007), with those practising for more than 5 years making the diagnosis more frequently than those practising for 5 years or less (59% *v*. 41%, *P* = 0.003), despite all but one being aware of the diagnostic features of brain fag. Similarly, seniority in clinical practice was associated with the diagnosis of brain fag (*P* = 0.003), with consultants diagnosing more than their junior colleagues. There was no difference in proportion of psychiatrists diagnosing brain fag when comparing respondents from different states and regions of Nigeria (*P*>0.05). Also, the level of specialisation of institutions was not associated with a difference in likelihood of diagnosing (general hospital 23% *v*. teaching hospital 20%, *P*>0.05). Using binary logistic regression analysis, the only independent predictor of diagnosing brain fag syndrome was the seniority of clinician (*P* = 0.008). Registrars were least likely to make a diagnosis compared with consultants (odds ratio 0.1, 95% CI 0.03-0.38, *P* = 0.001).

## Discussion

These important findings suggest that the less experienced psychiatrists, who are more likely younger, made a diagnosis of brain fag syndrome least. It is unclear whether they found brain fag less attractive than their senior colleagues or if this may reflect differences in training (postcolonial *v*. contemporary) and diagnostic emphasis between the groups (A. Ayonrinde, personal communication, 2012) Should this trend persist, we foresee decline and possible extinction in the use of this diagnosis among Nigerian psychiatrists, critically within the society in which this culture-bound syndrome was originally described.

### Symptom recognition and diagnostic instability

We observed diagnostic inconsistencies among Nigerian psychiatrists presented with the vignette with features of brain fag syndrome. Although familiar with the presentation (98%), and some consideration given to brain fag, the majority of psychiatrists preferred the more familiar ICD-10 diagnoses to a culture-bound syndrome. The phrase ‘brain fag’ was once a familiar idiom of distress during the earlier educational years of some senior clinicians, however, it is no longer common cultural parlance within Nigerian society. High rates of term recognition may also be a consequence of postgraduate psychiatric training where brain fag is taught as a disorder with a West African history. In fact, the authors (of Nigerian ancestry) with cultural and clinical experience of Nigerian mental health services have rarely heard use of the term brain fag outside academic circles.

The clinical and cultural consonance of the psychiatrists surveyed in this study enriches the findings as they span several decades of experience, clinical centres, ethnic and geographical regions. Their clinical and social awareness of emotional distress among students gives useful insight into the contemporary concept of brain fag.

Our finding of diagnostic fluidity and heterogeneity between anxiety, somatisation and depressive disorders is consistent with the DSM-IV-TR statement that brain fag syndrome can resemble anxiety, depressive and somatoform disorders.^7^

The diagnostic instability evident here calls into question whether brain fag syndrome is a homogenous or unique diagnostic entity, or actually a symptom co-variant of other somatisation, anxiety and affective disorders. The weight of cognitive and somatic complaints has been observed to differentiate from core features of depression in Nigeria and may well confirm unique culture-specific presentation^10^ as was proposed in the 1960s.^15^

Educational concerns, intensive or excessive study and the drive for success were the most common explanatory models for the vignette (32%). The emphasis on education and ‘study’ in the brain fag narrative may unwittingly bias clinicians towards cerebral and mental symptoms, thereby distracting from other somatic complaints. We hypothesise that brain fag was probably a historic idiomatic expression of impaired mental function with presentation of somatic complaints. Sleep impairment, poor attention and concentration and somatic complaints in an anxious or depressed individual would understandably impair educational activity – whereby symptoms worsen the potential to study rather than study triggering a mental disorder.

The brain fag explanatory models presented biological, psychological, social and stress models of illness. To date these aetiological links are lacking in robust empirical evidence. Further exploration of the somatic manifestations of common tropical diseases such as malaria would be insightful. Interestingly, one respondent cited ‘witchcraft’. Beliefs in evil spirits, curses and malevolent forces are not uncommon in West African cultures. Psychoactive substance use and their effects such as impaired concentration and sleep difficulties and somatic effects may also easily mimic the brain fag symptoms.

### Management

The preference for psychological therapies appears to reflect the aetiological attributions given to the symptoms such as stress, educational aspirations and interpersonal difficulties. To date there has been mainly anecdotal evidence or brief reports on treatments for brain fag.^16^ It is unclear from our findings what the response is to these therapeutic interventions and the degree of symptom relief. Given that the majority of psychiatrists were of the opinion that the symptom clusters were features of somatisation, anxiety and affective disorders, the additional choices of psychotropic medication are understandable.

Improving our understanding of the causes of brain fag symptoms has potential benefits in terms of identifying susceptible individuals and subsequently being able to manage them more appropriately. The fact that nearly a third of respondents pointed to educational concerns as a factor in the development of brain fag could have a significant influence on educational and public health interventions, policies as well as practice. Inaccurate information regarding causative factors and the care of mental distress in students carries a risk of prolonging mental distress. This confusion may result in individuals with depression or an anxiety disorder believing themselves to experience brain fag and abandoning education.

There is a need for improved understanding of mental disorders ascribed to study in this West African culture given the cultural salience of education as a key to success and social mobility. It remains unclear whether brain fag is a mental sequel of educational difficulties or the somatic, neurotic, cognitive and affective manifestation of distress in students. Significantly more research is required into cultural concepts of distress,^9^ an important consideration for the ICD-11.

Over half a century on from the original observations on brain fag it seems that our understanding of this culture-bound disorder has not advanced much. This study found that a large proportion of psychiatrists in Nigeria were familiar with and regularly manage students who present with anxiety, affective, cognitive and somatic symptoms. However, these African psychiatrists in the home of the brain fag syndrome infrequently consider this diagnosis in their routine clinical practice.

The semiotic salience of brain fag as a distinct, relevant and contemporary culture-bound syndrome is not supported by our findings in Nigeria. Idiomatic and syndromic use of the term brain fag became extinct in 19th- and 20th-century Britain, before its resurgence in Africa.^17,18^ Should the modern decline in brain fag persist, this culture-bound syndrome may well face diagnostic extinction.

## References

[1] Prince R The ‘brain fag’ syndrome in Nigerian students. J Ment Sci 1960; 106: 559-70. 14434928 10.1192/bjp.106.443.559

[2] Harris B A case of brain fag in East Africa. Br J Psychiatry 1981; 139: 162-3. 6118193 10.1192/bjp.139.2.162

[3] Peltzer K Cherian VI Cherian L Brain fag symptoms in rural South African secondary school pupils. Psychol Rep 1998; 83 (3 Pt 2): 1187-96. 10079714 10.2466/pr0.1998.83.3f.1187

[4] Jegede RO Psychiatric illness in African students: ‘brain fag’ syndrome revisited. Can J Psychiatry 1983; 28: 188-92. 6850499 10.1177/070674378302800306

[5] Ola BA Morakinyo O Adewuya AO Brain fag syndrome a myth or Reality. Afr J Psychiatry 2009; 12: 135-43. 10.4314/ajpsy.v12i2.43731 19582315

[6] Aina OF Morakinyo O Culture-bound syndromes and the neglect of cultural factors in psychopathologies among Africans. Afr J Psychiatry 2011; 14: 278-85. 10.4314/ajpsy.v14i4.4 22038425

[7] World Health Organization The ICD-10 Classification of Mental and Behavioural Disorders. WHO, 1992.

[8] American Psychiatric Association *Diagnostic and Statistical Manual of Mental Disorders Fourth Edition, Text Revised* (DSM-IV-TR). APA, 2000.

[9] American Psychiatric Association *Diagnostic and Statistical Manual of Mental Disorders Fifth Edition* (DSM-5). APA, 2013.

[10] Okulate GT Olayinka MO Jones OBE Somatic symptoms in depression: evaluation of their diagnostic weight in an African setting. Br J Psychiatry 2004; 184: 422-7. 15123506 10.1192/bjp.184.5.422

[11] Ola BA Igbokwe DO Factorial validation and reliability analysis of the brain fag syndrome scale. Afr Health Sci 2011; 11: 334-40. 22275921 PMC3261013

[12] World Health Organization WHO-AIMS Report on Mental Health System in Nigeria. WHO, 2006.

[13] Reed GM Mendonça Correia J Esparza P Saxena S Maj M The WPA-WHO Global Survey of Psychiatrists' Attitudes Towards Mental Disorders Classification. World Psychiatry 2011; 10: 118-31. 21633689 10.1002/j.2051-5545.2011.tb00034.x PMC3105474

[14] Kellerman SE Herold J Physician response to surveys: a review of the literature. Am J Prevent Med 2001; 20: 61-7. 10.1016/s0749-3797(00)00258-0 11137777

[15] Mbanefo SE ‘Heat in the body’ as a psychiatric symptom: its clinical appraisal and prognostic indication. J Coll Gen Pract 1966; 11: 235-40. 4379829 PMC2237841

[16] Anumonye A Lorazepam in the treatment of ‘brain fag syndrome’. Afr J Psychiatry 1978; 3: 121-3.

[17] Ayonrinde OA Bhugra D >Culture bound syndromes. In Troublesome Disguises: Managing Challenging Disorders in Psychiatry (2nd edn) (eds Bhugra D Malhi GSA). Wiley-Blackwell, 2015.

[18] Ayonrinde OA Brain fag syndrome: new wine in old bottles or old wine in new bottles? Niger J Psychiatry 2008; 6: 47-50.

